# Electron-Beam-Initiated Crosslinking of Methacrylated Alginate and Diacrylated Poly(ethylene glycol) Hydrogels

**DOI:** 10.3390/polym15244685

**Published:** 2023-12-12

**Authors:** Arn Mignon, Joanne Zimmer, Carolina Gutierrez Cisneros, Mathias Kühnert, Elien Derveaux, Olesya Daikos, Tom Scherzer, Peter Adriaensens, Agnes Schulze

**Affiliations:** 1 Smart Polymeric Biomaterials, Biomaterials and Tissue Engineering, Campus Group T, KU Leuven, Andreas Vesaliusstraat 13, 3000 Leuven, Belgium; caro.cisneros@kuleuven.be; 2 Department of Surfaces of Porous Membrane Filters, Leibniz Institute of Surface Engineering (IOM), Permoserstr. 15, 04318 Leipzig, Germany; joanne.zimmer@pkm.tu-darmstadt.de (J.Z.); mathias.kuehnert@iom-leipzig.de (M.K.); agnes.schulze@iom-leipzig.de (A.S.); 3 Institute for Condensed Matter Physics, Technische Universität Darmstadt, Hochschulstraße 8, 64289 Darmstadt, Germany; 4 Analytical and Circular Chemistry (ACC), NMR Group, Institute for Materials Research (IMO-IMOMEC), Hasselt University, Agoralaan-Building D, 3590 Diepenbeek, Belgium; elien.derveaux@uhasselt.be (E.D.); peter.adriaensens@uhasselt.be (P.A.); 5 Leibniz Institute of Surface Engineering (IOM), Material Characterization and Analytics, Permoserstr. 15, 04318 Leipzig, Germany; olesya.daikos@iom-leipzig.de (O.D.); tom.scherzer@iom-leipzig.de (T.S.)

**Keywords:** alginate, poly(ethylene glycol), (meth)acrylates, electron beam, physicochemical characterization, transparency, rheology

## Abstract

An ideal wound dressing not only needs to absorb excess exudate but should also allow for a moist wound-healing environment as well as being mechanically strong. Such a dressing can be achieved by combining both a natural (alginate) and synthetic (poly(ethylene glycol) polymer. Interestingly, using an electron beam on (meth)acrylated polymers allows their covalent crosslinking without the use of toxic photo-initiators. The goal of this work was to crosslink alginate at different methacrylation degrees (26.1 and 53.5% of the repeating units) with diacrylated poly(ethylene glycol) (PEGDA) using electron-beam irradiation at different doses to create strong, transparent hydrogels. Infrared spectroscopy showed that both polymers were homogeneously distributed within the irradiated hydrogel. Rheology showed that the addition of PEGDA into alginate with a high degree of methacrylation and a polymer concentration of 6 wt/v% improved the storage modulus up to 15,867 ± 1102 Pa. Gel fractions > 90% and swelling ratios ranging from 10 to 250 times its own weight were obtained. It was observed that the higher the storage modulus, the more limited the swelling ratio due to a more crosslinked network. Finally, all species were highly transparent, with transmittance values > 80%. This may be beneficial for the visual inspection of healing progression. Furthermore, these polymers may eventually be used as carriers of photosensitizers, which is favorable in applications such as photodynamic therapy.

## 1. Introduction

Wound healing is a dynamic process that may give rise to the development of large amounts of blood and exudate. Hemostatic agents are effective alternatives for the controlling of bleeding. Hydrogel-based dressings cover the injury site, absorb the blood and exudate and provide physical support during the wound-healing process [1]. The ideal wound dressing should not only absorb excess exudate but also create a moist wound-healing environment, be mechanically strong and be easily removed [2,3].

Alginate is a natural polymer that has already proven its suitability for wound-healing applications [4,5,6,7]. Due to its high water-uptake capacity, hemostatic properties and biocompatibility, alginate provides an environment that promotes tissue regeneration [8]. Nonetheless, the performance of sodium alginate in its ionically crosslinked form (calcium alginate) is still mechanically weak. Alginate benefits, however, from different functional groups that allow modifications to increase its mechanical properties. One such possibility is the incorporation of methacrylate functionalities using, for example, methacrylic anhydride to create methacrylated alginate (AlgMA) [9]. Depending on the degree of methacrylation, different physicochemical and mechanical properties are obtained. Indeed, incorporating functional groups that can be covalently crosslinked allows for a stronger network compared with ionic crosslinks through calcium ions. 

However, further enhancement of the mechanical strength is often necessary by the addition of synthetic polymers in the form of crosslinkers or as blends. Poly(ethylene glycol)-based hydrogels have already frequently been used in different biomedical applications, including tissue engineering, drug release, and wound healing [1,10,11,12]. The incorporation of acrylate end groups into poly(ethylene glycol) diacrylate (PEGDA) also allows covalent crosslinking, similar to AlgMA. Indeed, (meth)acrylate groups can be photocrosslinked with one another using a photo-initiator (PI). However, the leftovers of these PIs can result in toxic effects, which could be detrimental when applied as wound dressings [13,14]. 

To avoid the addition of toxic PIs, an electron beam (E-beam) offers a very interesting alternative, given that electrons emitted from a linear accelerator initiate a polymerization reaction. Glass et al. recently showed that poly(ethylene glycol) diacrylate (PEGDA) hydrogels polymerized via an electron beam displayed increased mechanical stability and greater transparency compared with conventional PEGDA hydrogels polymerized using a combination of a PI and UV light [12]. In addition, electron-beam irradiation sterilizes the sample, which is advantageous for medical applications [15]. 

An additional benefit of alginate- and PEG-based polymers is their transparency [16]. This enables a visual follow-up of the wound-healing process. This transparency opens up further possible applications that progress beyond a passive wound dressing, i.e., a transparent hydrogel can act as a carrier system of photo-active drugs for photodynamic therapy [12]. In this regard, the effective light-induced activation of photosensitizers requires high hydrogel transparency. Glass et al. showed that photosensitizer-loaded PEGDA hydrogels were efficient in the light-induced eradication of seven multi-resistant bacterial strains [17]. By this, they demonstrated that they possess great potential for the treatment of wounds infected by multi-resistant bacteria.

The goal of this work was to crosslink AlgMA at different methacrylation degrees with PEGDA using E-beam irradiation at different doses. Subsequently, the effect of these parameters on the crosslinking efficiency, swelling ratio and mechanical strength were determined. Finally, as a proof-of-concept, the transparency was measured as a potential opportunity for future controlled release.

Combining the strong swelling properties of methacrylated alginate and the mechanical strength of poly(ethylene glycol)diacrylate and processing them into a homogeneous covalently crosslinked network using E-beam irradiation without the need of a possible toxic photo-initiator opens the door to multiple biomedical applications such as wound healing. 

## 2. Materials and Methods

### 2.1. Materials

Poly(ethylene glycol) diacrylate (PEGDA) (average molecular weight 700 g mol^−1^) was purchased from Sigma Aldrich (St. Louis, MI, USA). Ultrapure water was generated using a Merck ultrapure water system (Darmstadt, Germany). Sodium alginate (Na Alg), methacrylic anhydride (MAAH) and sodium hydroxide were obtained from Sigma-Aldrich. Dialysis membranes (Spectra, Por 4; 12–14 kDa) were bought from VWR. 

### 2.2. Synthesis of Methacrylated Alginate

**Figure 1:**
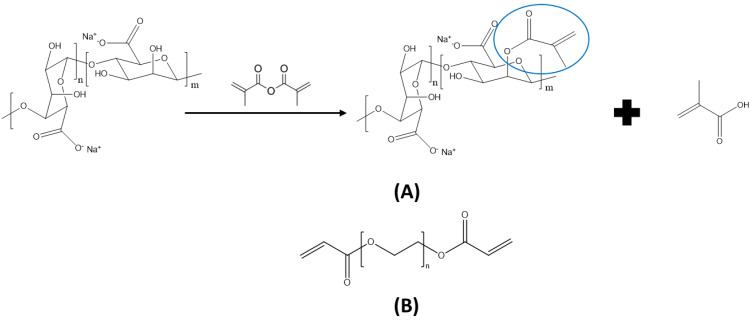
(**A**) Synthesis of methacrylated alginate, starting from Na Alg, with methacrylic acid as side product. (**B**) Poly(ethylene glycol diacrylate).

To methacrylate alginate, the hydroxyl functionalities were modified using MAAH. This introduced methacrylate groups, which allowed for covalent crosslinking (Figure 1A). In both the low and high modification degrees, MAAH was dropwise added to a 2 wt% Na Alg solution (2 or 4 equivalent MAAH per hydroxyl group in alginate for a low and high degree of methacrylation, respectively). As a side product, the formation of methacrylic acid continuously decreased the pH, so 5 M of a NaOH solution was added dropwise to maintain a pH of 8. The reaction was performed for 24 h at room temperature, after which the modified polymer was purified by dialysis (12–14 kDa membranes). The water was changed three times per day for 72 h. Finally, the material was dried by lyophilization.

### 2.3. Degree of Methacrylation

The degree of methacrylation (i.e., the amount of incorporated double bonds), which influenced the mechanical and swelling properties of the formed hydrogels, was determined using ^1^H-NMR spectroscopy (similar to our previous research [18]). In brief, the vinyl protons at 5.75 and 6.15 ppm corresponded with the two positions of potentially attached methacrylate groups, which were compared with the reference protons in the alginate backbone at 4.97 ppm. This corresponded with the anomeric carbon of guluronic acid blocks (G-units). As there were also mannuronic acid moieties present (M-units), with a reference peak at 4.58 ppm, the G-proportion needed to be determined and taken into account as a correction factor. The percentage of G-units was calculated using Equation (1). In a subsequent step, the degree of substitution (DS) per repeating unit was determined from the average of the intensity of the methacrylate groups’ peaks (see Equation (2)). As every repeating unit had two hydroxyl groups, the DS (as a function of the hydroxyl groups) could be calculated by dividing the previously calculated value by two.

(1) $$G\left[\%\right]=\frac{I_{4.97ppm}}{I_{4.58ppm}+I_{4.97ppm}}*100\%$$

(2) $$DS\left[\%\right]=G\;*\;\frac{\frac{I_{5.75ppm}+I_{6.15ppm}}{2}}{I_{4.97ppm}}$$

### 2.4. Hybrid Hydrogel Synthesis

**Figure 2:**
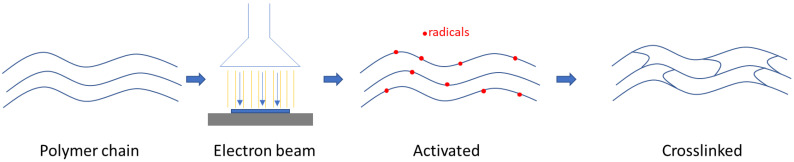
Schematical representation of electron-beam crosslinking of the polymer solutions.

In the first step, AlgMA was dissolved in ultrapure H_2_O (see Table 1 for the final concentrations) using a speed mixer (DAC 150.1 FVZK, Synergy Devices Limited, High Wycombe, UK). Secondly, PEGDA (Figure 1B) was added and homogenized using the same device. The solutions were then flushed with argon (Ar) for 15 min. In the next step, 3.0 mL of the solution was pipetted into polystyrene molds with a diameter of 35 mm and then stored under an Ar atmosphere until the E-beam crosslinking using a 10 MeV linear accelerator (MB1030 MP, Mevex Corp, Stittville, ON, Canada). The crosslinking process is described in Figure 2. The synthesis of pure AlgMA and PEGDA reference hydrogels, respectively, was carried out in a similar manner.

### 2.5. Infrared Spectroscopy

The absorption spectra were recorded using a Bruker Fourier-transform infrared (FTIR) spectrometer with a Golden Gate single-reflection diamond-attenuated total reflection system (Specac, Kent, UK). The sample was directly positioned on the attenuated total reflectance (ATR) diamond and 16 scans were recorded for each sample. Before the measurement, hydrogel samples with a diameter of 1 cm were dried in the oven for 48 h.

### 2.6. Infrared Microspectral Imaging

A Hyperion 3000 Infrared microscope coupled to a Tensor II FTIR spectrometer (Bruker Optik) was used for microspectral imaging in ATR mode using an objective with a germanium crystal. The images were recorded using a 64 × 64 pixel focal plane array (FPA) detector. Each ATR image covered a size of 32 × 32 µm. Considering the angle of incidence of the infrared radiation in the ATR crystal (Θ = 29°) as well as the refractive indices of germanium (n_D_ = 4.0), PEGDA (n_D_ = 1.47) and AlgMA (n_D_ = 1.5), the penetration depth of the probe light into the sample (defined as the decay of the field strength to 1/e) was about 800 nm at the position of the alginate band at 1607 cm^−1^, which was used for the generation of spectral images. The spectral resolution was set to 8 cm^−1^. Spectra with 64 scans were recorded in order to obtain an adequate signal-to-noise ratio. Several images were taken from various positions of both the top and the bottom side of each hydrogel sample.

### 2.7. Rheology

The viscoelastic properties—i.e., the storage modulus (G′) and loss modulus (G″) of the hydrogels—were determined using an MCR-300 bulk rheometer (Anton Paar, Graz, Austria) in rotational oscillation at 25 °C. Using 10 mm parallel-plate geometry, a frequency sweep with a 1% strain in a region from 0.01 Hz to 10.00 Hz was performed. For data evaluation, the value at 1 Hz was utilized. The mean values of G′ and G″ were obtained by measuring at least four individual hydrogel samples of the same composition. 

The hydrogel crosslinking density ν_c_ was determined using Equation (3):

(3) $$\nu_{c}={(G}^{\prime }/RT)$$

G′ refers to the storage modulus at 1 Hz determined by rheology, R is the universal gas constant (8.314 J mol^−1^ K^−1^) and T is the temperature during the measurement (298 K).

### 2.8. Swelling

For the determination of the gel fraction and the swelling ratio, the samples (n = 4; diameter of 1 cm) were first dried in an oven at 40 °C for 24 h (w_0_), then they were immersed in ultrapure H_2_O and left to swell for 72 h at 25 °C. The swollen samples were then removed from the solution, dabbed dry to remove the excess H_2_O and weighed (w_S_). Subsequently, the samples were again dried in an oven for 48 h and weighed after that (w_1_). The calculations of the gel fraction and swelling ratio were ascertained according to Equations (4) and (5):

(4) $$Gel\;fraction\left[\%\right]=\frac{w_{1}}{w_{0}}\;*\;100\%$$

(5) $$Swelling\;ratio[/]=\frac{w_{S}-w_{1}}{w_{1}}$$

### 2.9. UV–Vis 

The transmittance spectra in a wavelength range from 200 to 800 nm were obtained using a Cary 5000 UV–VIS-NIR spectrometer from Agilent (Santa Clara, CA, USA) with a step width of 0.5 nm. Directly after E-beam irradiation, the samples were placed on the holder for the measurement of their optical transmittance.

### 2.10. Statistics

The mean and standard deviation (SD) were calculated (n ≥ 3) and were expressed as mean ± SD. A statistical evaluation was carried out using a one-way ANOVA. Differences were considered significant when *p* < 0.05.

## 3. Results

The methacrylation of alginate was successfully performed, creating two batches with a high (53.5% of the repeating units) and low (23.3% of the repeating units) DS (see Supplementary Materials Figures S1 and S2). For ease of reading, the prepared samples were given codes; these are explained in Table 1.

After E-beam crosslinking, the samples were analyzed to measure the effect of the E-beam on the physicochemical and mechanical properties. In the first step, the samples were analyzed using FTIR to confirm the crosslinking of both polymers. Additionally, infrared mapping was performed using microspectral imaging to identify the polymer homogeneity. The rheology measurements indicated the storage modulus of the formed hydrogels and the crosslinking density of the gels. The crosslinking density could also be linked to the results obtained for the swelling ratio and the gel fraction. The latter provided a qualitative measure of the crosslinking efficiency. Finally, the optical transmittance was measured to assess the potential for photosensitizer release.

### 3.1. Confirmation of the Presence of Both AlgMA and PEGDA in the Hydrogels

**Figure 3:**
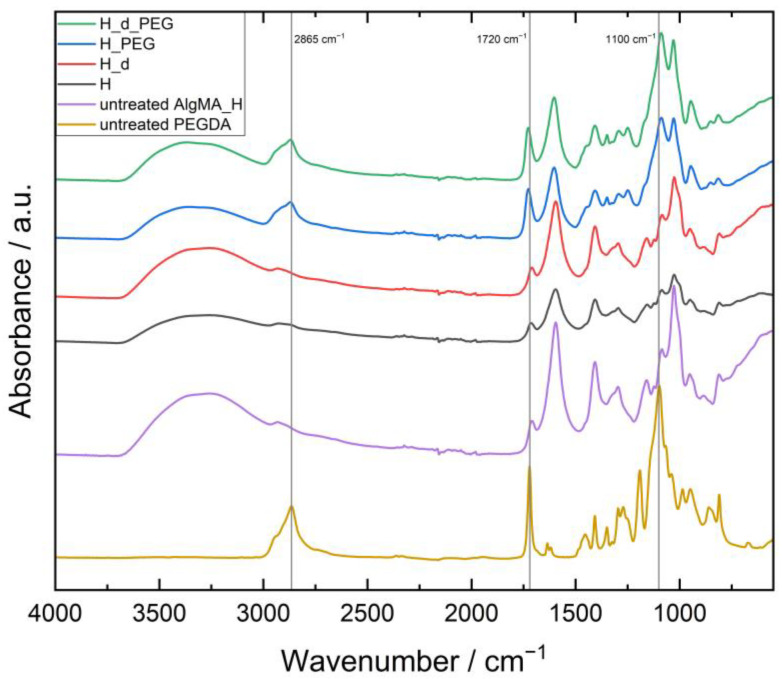
FTIR spectra of unirradiated AlgMA H and PEGDA as well as H, H_d, H_PEG and H_d_PEG irradiated with 1 × 5 kGy.

**Figure 4:**
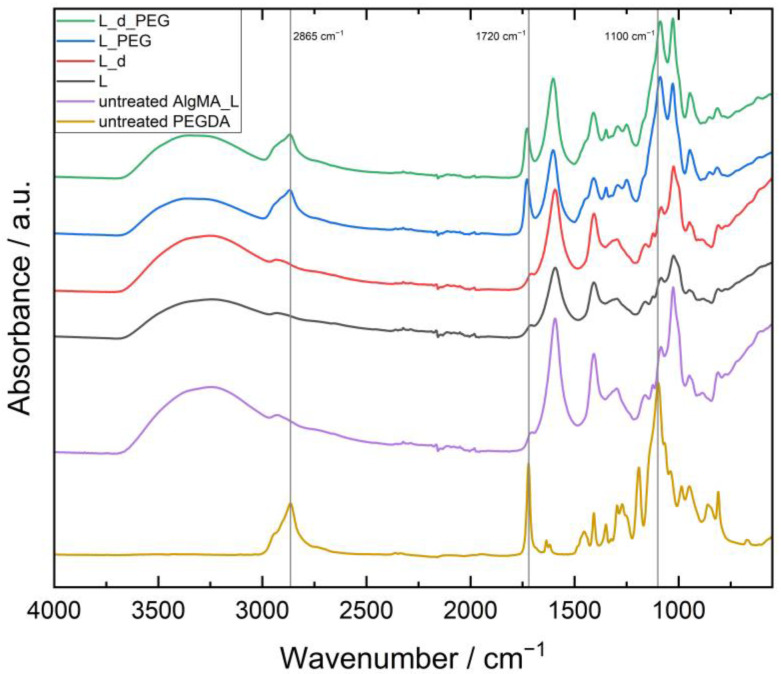
FTIR of unirradiated AlgMA L and PEGDA as well as L, L_d, L_PEG and L_d_PEG irradiated with 1 × 5 kGy.

To confirm that both materials were present and crosslinked after E-beam irradiation, ATR–FTIR was performed on the samples. As an example, the spectra of H_1x5, H_d_1x5, H_PEG_1x5 and H_d_PEG_1x5 were compared with untreated H and PEGDA, as shown in Figure 3. A strong peak at 1100 cm^−1^ linked to the C-O stretch of the ether groups of PEG was also strongly visible in the H_PEG and H_d_PEG gels, similar to the C-H stretch at 2865 cm^−1^. At 1720 cm^−1^, the C=O stretch vibration was noticed from the conjugated ester functionalities [19]. For the low molecular weight PEGDA, this was logically much more present than for the alginate, where only a part of the alcohol moieties was modified in the entire polymer. The broad O-H stretch vibration ν(O-H) of the carboxylic acid moieties of alginate was spread between 2400 and 3400 cm^−1^. A similar reasoning could be used for the L hydrogels seen in Figure 4. The difference between untreated AlgMa L and AlgMA H was especially visible at 1720 cm^−1^ for the C=O stretch vibration of the ester groups introduced after methacrylation, which was clearly more visible in the untreated AlgMA H. To determine the homogeneity of the networks, FTIR microspectral imaging was performed. 

### 3.2. Homogeneity of the Crosslinked Networks Studied by FTIR Microspectral Imaging

Microspectral imaging was used to study the homogeneity of the polymer networks after crosslinking using E-beam irradiation. As the samples were too thick for infrared measurements in transmission, the ATR mode was chosen. The refractive index of the germanium crystal in the objective was very high, so the penetration depth of the incident infrared radiation into the sample was less than 1 µm, which indicated that the characterization was limited to a thin surface layer. Therefore, the samples were investigated using various positions from the top and bottom sides of the dry hydrogel samples.

**Figure 5:**
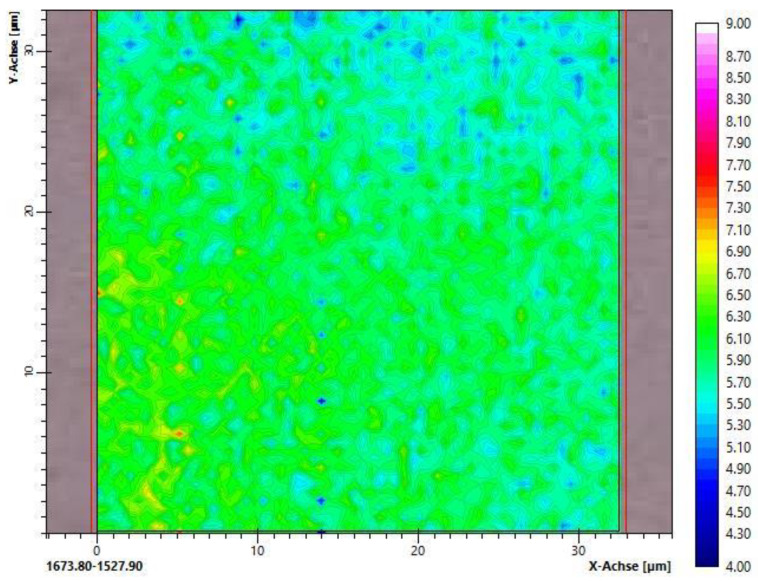
Spectral image of H_PEG after irradiation with an electron dose of 1 × 3 kGy.

At first, the spectral images of the crosslinked hydrogels consisting of the methacrylated alginates H_PEG and L_PEG were recorded. This also comprised samples that were irradiated at different electron irradiation doses (3 or 5 kGy). An example is shown in Figure 5, where high homogeneity can be confirmed. In particular, no phase separation occurred. The same applied to all other hydrogels containing PEG. Their spectral images are given in the Supplementary Materials (Figure S3).

Absorption bands of C=C in the (meth)acrylate group appeared at 1640, 1410, 940 and 810 cm^−1^. It was clear from the IR spectra of the methacrylated alginate before irradiation (Figure S4) that all these bands were hidden by the strong absorption bands of the alginate. The only bands related to the methacrylate that could clearly be identified in those spectra were the carbonyl band at 1710 cm^−1^ and the C-O stretching band at 1160 cm^−1^. It is well known that the latter band disappears during the conversion of the (meth)acrylic double bond, which is probably related to a change in the force constant due to the conversion of the double bond in the proximity, leading to a shift to another wavenumber. A detailed analysis of the spectra showed that the (meth)acrylic bonds of PEGDA and the modified alginate were completely converted, even after irradiation with only 3 kGy. 

### 3.3. Rheology

After the confirmation of successful and homogeneous crosslinking using E-beam irradiation with FTIR microspectral imaging, the mechanical properties were investigated using rheology. This provided the storage (elastic) modulus G′ and loss (viscous) modulus G″. G′ characterizes stored deformation energy, while G″ is a representation of the lost deformation energy through internal friction [20]. In all cases, G′ > G″ was observed, which proved that hydrogels were successfully formed. To ensure clarity, only the storage modulus is represented. For research purposes, the loss modulus can be found in the Supplementary Materials (Figures S5 and S6). Additionally, the crosslinking densities (mmol L^−1^) can be calculated from the storage modulus using Equation (3) (see Table 2).

**Figure 6:**
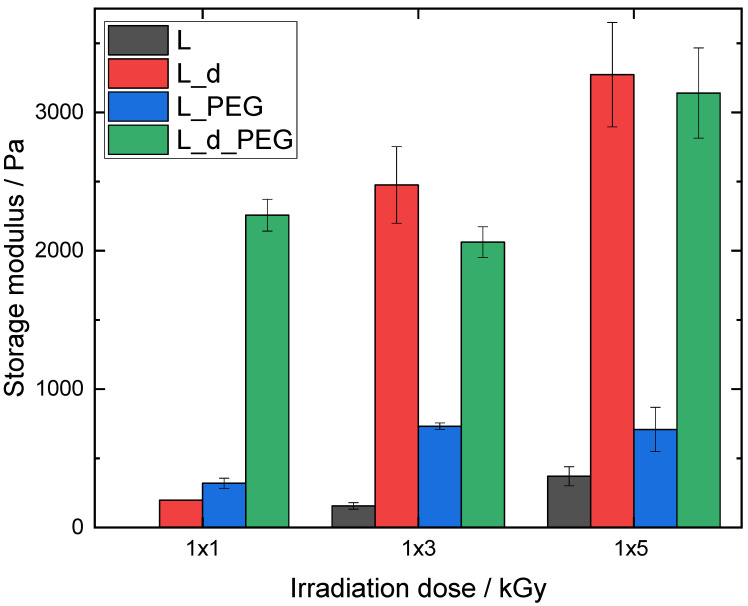
Storage modulus of AlgMA L hydrogels.

The samples with a low DS (L) were first measured with irradiation doses of 1 × 1, 1 × 3 and 1 × 5 kGy (Figure 6). A general trend for L and L_d was that G′ increased with an increase in irradiation dose. L_1x1 could not be measured as this sample did not lead to a hydrogel and remained liquid after E-beam irradiation. This was also the reason why L_d_1x1 had a much lower G′ compared with L_d_1x3 and L_d_1x5, as a strong network had not been formed at this low dose.

The hybrid material L_PEG showed a significantly higher G′ than L, meaning that PEGDA in the hybrid hydrogel was responsible for an increase in mechanical stability. There was a significant difference (*p* < 0.05) between L_PEG_1x1 and L_PEG_1x3, but no further significant increase (*p* > 0.05) compared with L_PEG_1x5. The concentration of two equivalents led to a strong rise in G′, indicating increased mechanical stability due to denser crosslinking. This increase in mechanical strength was only significant when comparing the dose of 1 × 3 with 1 × 5 kGy. To further enhance the mechanical properties and stability of the irradiated polymers, a higher degree of methacrylation was also investigated (H hydrogels), whereby similar irradiation doses were employed as for the L series (1 × 1, 1 × 3 and 1 × 5 kGy), as well as the effect of multiple 5 kGy doses (2 × 5 and 4 × 5 kGy).

**Figure 7:**
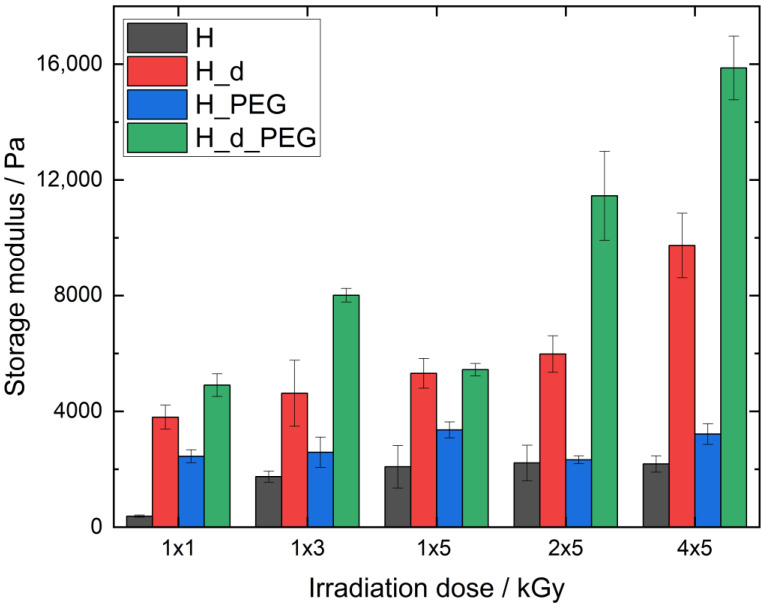
Storage moduli of AlgMA H hydrogels.

Figure 7 represents the G′ moduli of different H hydrogels with the varying E-beam doses. As expected, the G′ of H was the lowest for all four types of investigated materials; at 1 × 1 kGy, G′ was as low as 378 ± 37 Pa. From an irradiation dose of 1 × 3 kGy or higher, G’ did not significantly change (*p* < 0.05), which indicated that the maximum number of crosslinking junctions was achieved, indicating that the material could not gain further stability with a higher irradiation dosage. It also showed that H remained stable at high irradiation doses. Interestingly, all values were significantly higher (*p* < 0.05) than those of the L hydrogels (see Figure 6).

The hybrid material H_PEG exhibited higher G′ values than H (significantly higher (*p* < 0.05), except for 2 × 5 kGy), but with a similar trend as for H. The main difference was the G’ treated with 1 × 1 kGy, which was already high (2448 ± 37 Pa) and not significantly different from the other doses of H_PEG, indicating that, similar to L_PEG, the PEGDA proportion in the hybrid hydrogel was responsible for the increase in mechanical stability at a dose of 1 × 1 kGy.

The samples prepared with a double total concentration (H_d and H_d_PEG) led to a strong significant increase in G′ in both cases compared with the lower concentrations. Also, the effect of additional PEGDA produced a significantly higher G′ when comparing H_d_PEG with H_d (*p* < 0.05) at all doses, except for H_d_PEG_1x5. This specific measurement was unexpected and requires further investigation in future work. For H_d, doubling the dose led to an increase in G’ and stability, with the highest value at 4 × 5 kGy (9735 ± 1115 Pa), although this was not significantly different from 1 × 1–2 × 5 kGy. Interestingly, the G′ of H_d_PEG rose significantly from 1 × 1 kGy and was highest at 4 × 5 kGy (15,867 ± 1102 Pa). In this trend, 1 × 5 kGy was not taken into account due to its unexpected results. The storage moduli also allowed for the calculation of the crosslinking densities, which are described in Table 2.

### 3.4. Physicochemical Characterization of the Crosslinked Hydrogels

In addition to the mechanical properties, it was also important to explore the swelling ratio of the formed hydrogels and to determine the crosslinking efficiency by measuring the gel fraction. As expected from the rheology measurements, the material composition and irradiation dose significantly influenced the hydrogel’s swelling properties and gel fractions. The same radiation doses were used for the rheological measurements to allow for a comparison.

Due to its carboxylate moieties and strong hydrophilic structure, the swelling potential of alginate is significantly higher (*p* < 0.05) than PEGDA [21]. There was a clear trend observed when comparing H and L with H_PEG and L_PEG, respectively. A similar tendency was seen for the samples with a double initial concentration. However, it was not statistically significant for H_d_2x5 and H_d_PEG_2x5. 

**Figure 8:**
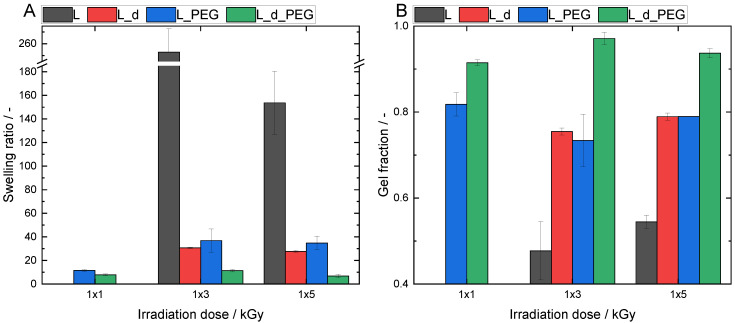
(**A**) Swelling ratios and (**B**) gel fractions for different variations of AlgMA L.

A comparable behavior was found for AlgMA L; however, in general, it showed higher swelling ratios (Figure 8). This was linked to the lower amount of methacrylate groups present and, thus, less densely crosslinked networks. At 1 × 3 kGy, the swelling ratio of L rose to 252 ± 19 its own weight. However, the network of this material was less stable, as confirmed by its low gel fraction. 

**Figure 9:**
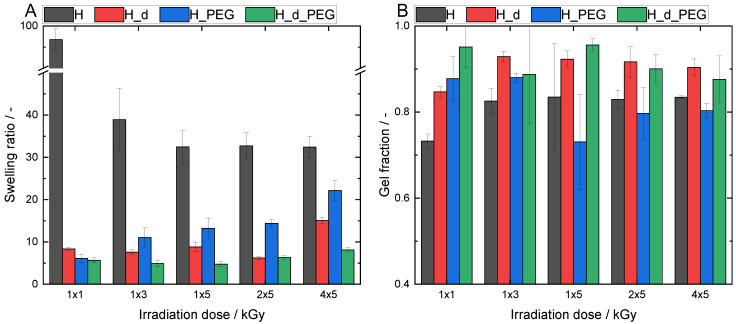
(**A**) Swelling ratios and (**B**) gel fractions for different variations of AlgMA H.

The hybrid material L_PEG, as well as the samples generated using higher concentrations (L_d and L_d_PEG), showed increased swelling ratios compared with their H counterparts (Figure 9).

A lower degree of methacrylation in AlgMA L led to a lower number of possible crosslinking junctions and thus, a less stable network. This achieved higher swelling ratios but correlated with lower gel fractions. This was the reason why a higher degree of methacrylation was anticipated to have significantly higher gel fractions but limited swelling ratios. 

When comparing all AlgMA H gels, the low concentration AlgMA H samples without PEGDA showed the highest swelling ratios for all irradiation doses. 

This trend was most pronounced at the lowest irradiation dose of 1 × 1 kGy, where AlgMA H had a swelling ratio of nearly 100 times its own weight (97 ± 2), while the three other materials (H_d, H_PEG and H_d_PEG) only swelled 6–8 times their own weight.

The swelling ratio for AlgMA H firstly decreased with an increase in irradiation dose and then appeared to reach a plateau. This was contrary to the swelling ratio of the hybrid material H_PEG, which increased with a rising irradiation dose. At first, this appeared to be counterintuitive, as an increased dose should lead to a denser network, thus decreased swelling. However, this correlated with the pore structure of the polymer and the chemical character of the solvent. Water uptake should be seen as the combination of swelling and the filling of pores [22]. An increase in porosity leads to an increased water-uptake capacity. Demeter et al. found that collagen/PVP/PEO hydrogels crosslinked with various irradiation doses had significant changes in swelling. An increased dose led to increased swelling [23]. This was due to the porosity and also the ionic properties of the hydrogels in a dense network. 

Conversely to AlgMA H, the hybrid material showed the highest swelling ratio of 22 ± 2 its own weight at the highest irradiation dose (4 × 5 kGy). When the alginate amount increased to 2 equivalents (hydrogel H_d), a strong decrease in the swelling ratio was observed compared with hydrogel H, e.g., up to a factor of 16 for 1 × 1 kGy. The swelling ratio of H_d slightly increased for the highest irradiation dose of 4 × 5 kGy. This could be explained by the study of Demeter et al. [23]. The hybrid material H_d_PEG showed the lowest swelling ratios for all irradiation doses, with no significant differences (*p* > 0.05). 

The hydrogel gel fractions showed a negative correlation with the swelling ratios and a positive correlation with the network strength. This might have produced more rigid hydrogels with lower swelling capacities. The gel fraction of H increased from an irradiation dose of 1 × 1 kGy (0.7 ± 0.02 %) to 1 × 3 kGy (0.8 ± 0.02 %), but then remained constant. This indicated that the maximum crosslinking had already been reached at 1 × 3 kGy, which was also linked to a stable swelling ratio with a further increase in dose. The gel fractions of H_PEG ranged between 80 and 90%, depending on the irradiation dose. It was concluded that the gel fractions of H_d and H_d_PEG were, in general, significantly higher, especially for H_d. This was expected because there was a higher concentration of crosslinkers, which could more easily form a higher number of crosslinking junctions.

### 3.5. Transparency of the Samples

**Figure 10:**
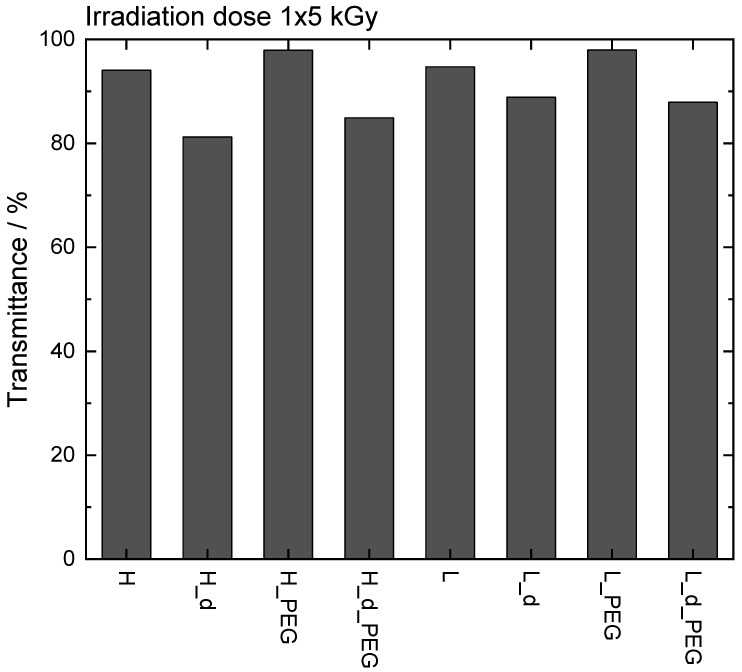
Transmittance of samples at 660 nm (absorption maximum of model drug methylene blue).

As a final test and proof-of-concept, we measured the transparency of the samples. This was ascertained after an irradiation dose of 1 × 5 kGy. Interestingly, all were highly transparent (see Figure 10), especially the polymers with a lower concentration. The UV–VIS curves can be found in the Supplementary Materials (Figure S7). This was logical as the double concentrations led to denser networks and thus, slightly lower transparencies. No statistical differences were tested as this was a proof-of-concept study on one sample. As an example, the transparent samples can be seen in the Supplementary Materials (Figure S8).

Transparent hydrogels are ideal carriers for photosensitizers, which are useful for photodynamic therapy, as was investigated by Glass et al. on PEGDA-based polymers [17]. These photosensitizers can then be released and lead to singlet oxygen generation. This proof-of-concept was designed to explore the potential of the materials for such applications in future work. 

## 4. Conclusions

The goal of this study was to create crosslinked hydrogels based on AlgMA and PEGDA via E-beam irradiation. First, the alginate was methacrylated with a high and low degree of substitution of 53.5 and 26.1% of the repeating units, respectively. The formation of hydrogels was analyzed using FTIR spectroscopy, proving that both polymers were present within the irradiated hydrogel. Microspectral imaging showed that AlgMA and PEGDA were homogeneously mixed after E-beam irradiation. The samples were mechanically characterized using rheology and physicochemically characterized using gel fraction determination and swelling tests. Rheology showed that the addition of PEGDA to the hydrogels improved the storage modulus. Stronger hydrogels were formed, with alginate bearing a high degree of substitution (AlgMA H) compared with hydrogels formed from alginate with a low degree of substitution (AlgMA L). Doubling the concentration of polymers in the solution from 3 to 6 wt/v% also significantly enhanced the strength, where the strongest material was composed of alginate with a high degree of substitution in a double concentration in combination with the same ratio of PEGDA (H_d_PEG) with an irradiation dose of 4 × 5 kGy (15,867 ± 1102 Pa). The storage moduli also allowed for the calculation of the crosslinking densities. Similar trends were observed with the gel fraction and swelling tests. In general, the AlgMA H hydrogels had significantly higher gel fractions than the AlgMA L hydrogels. However, this stronger and more stable network led to a more limited swelling capacity, which was also irradiation-dose-dependent. Doubling the concentration further improved the gel fractions to values above 90%. As a final proof-of-concept, the transparency of all species treated with a 1 × 5 kGy dose was measured. The transmittance values ranged from 80 to 95%. This may be useful as a photosensitizer carrier, which could be applied to photodynamic therapy. It was possible to create highly homogeneous mixed hydrogels composed of AlgMA and PEGDA crosslinked using E-beam irradiation. Using E-beam irradiation allowed us to avoid the use of possibly toxic photo-initiators of classical photocrosslinking. The combination of high gel fractions, sufficient swelling and mechanical properties and transparency renders these polymers useful for wound-healing applications. Future research could include varying PEG’s molar mass and the type of modification to further optimize the physical properties. Furthermore, in vitro and in vivo hydrogel studies are required for an initial indication of their safe use as wound dressings. 

## Figures and Tables

**Table 1:** Code explanation for the Results and Discussion sections.

Abbreviation	Explanation
H or L	Indication of the high or low DS of AlgMA, respectively
d	Total polymer concentration was 3 wt/v% in ultrapure water except when ‘d’ was added, then double the concentration was used
PEG	If ‘PEG’ is present in the naming, it indicates that PEGDA was added at a 1/1 weight ratio compared with AlgMA. The total concentration remained the same. If PEGDA was added by itself without AlgMA, it remains as PEGDA throughout the manuscript
A × B	The numbers indicated here as A and B indicate the number of irradiation cycles and the intensity of the E-beam in kGy, respectively. The used values were 1 × 1, 1 × 3, 1 × 5, 2 × 5 and 4 × 5 kGy

**Table 2:** Crosslinking densities in mmol L^−1^ calculated from the storage modulus of G’.

Sample	1 × 1 kGy	1 × 3 kGy	1 × 5 kGy	2 × 5 kGy	4 × 5 kGy
L	No gel	62.72	149.15		
L_d	79.46	998.40	1320.11		
L_PEG	128.98	295.28	285.73		
L_d_PEG	910.66	832.00	1266.66		
H	152.78	703.92	841.75	894.52	880.41
H_d	1532.90	1866.37	2143.37	2412.30	3927.05
H_PEG	987.31	1043.78	1355.40	937.89	1296.91
H_d_PEG	1979.66	3232.20	2194.47	4617.86	6400.54

## Data Availability

All data is present inside the graphs. No additional data will be put online.

## Supplementary Materials

The following supporting information can be downloaded at: https://www.mdpi.com/article/10.3390/polym15244685/s1.
