# Supplementary material for: Electron-Beam-Initiated Crosslinking of Methacrylated Alginate and Diacrylated Poly(ethylene glycol) Hydrogels
Source: Polymers (Basel). 2023 Dec 12;15(24):4685. doi: 10.3390/polym15244685 (PMC10747465; doi:10.3390/polym15244685)
Supplement: Supplementary file 1 [file polymers-15-04685-s001.zip › polymers-2680369-supplementary.pdf]

Supporting Information

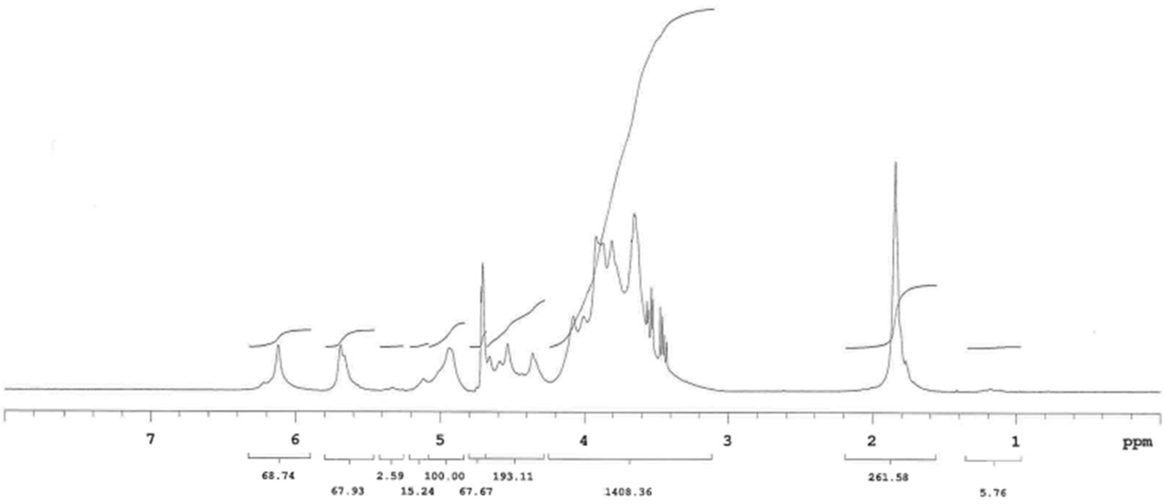

Figure S1. ^{1}H-NMR spectrum of the methacrylated alginate with a low degree of substitution (L hydrogels)

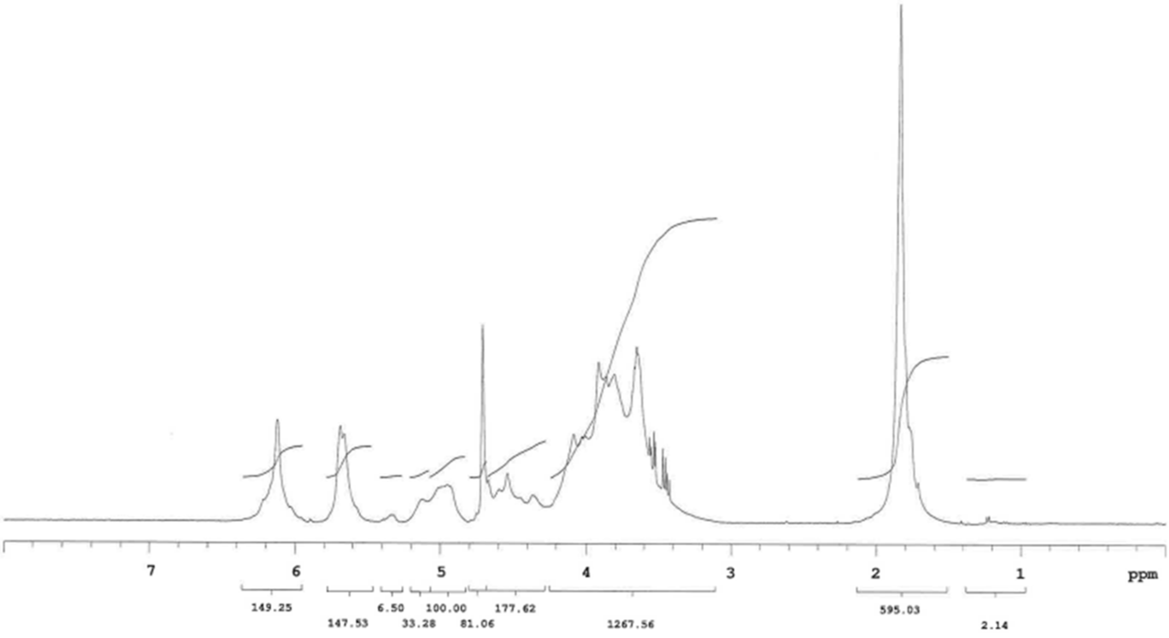

Figure S2. ^{1}H-NMR spectrum of the methacrylated alginate with a high degree of substitution (H hydrogels)

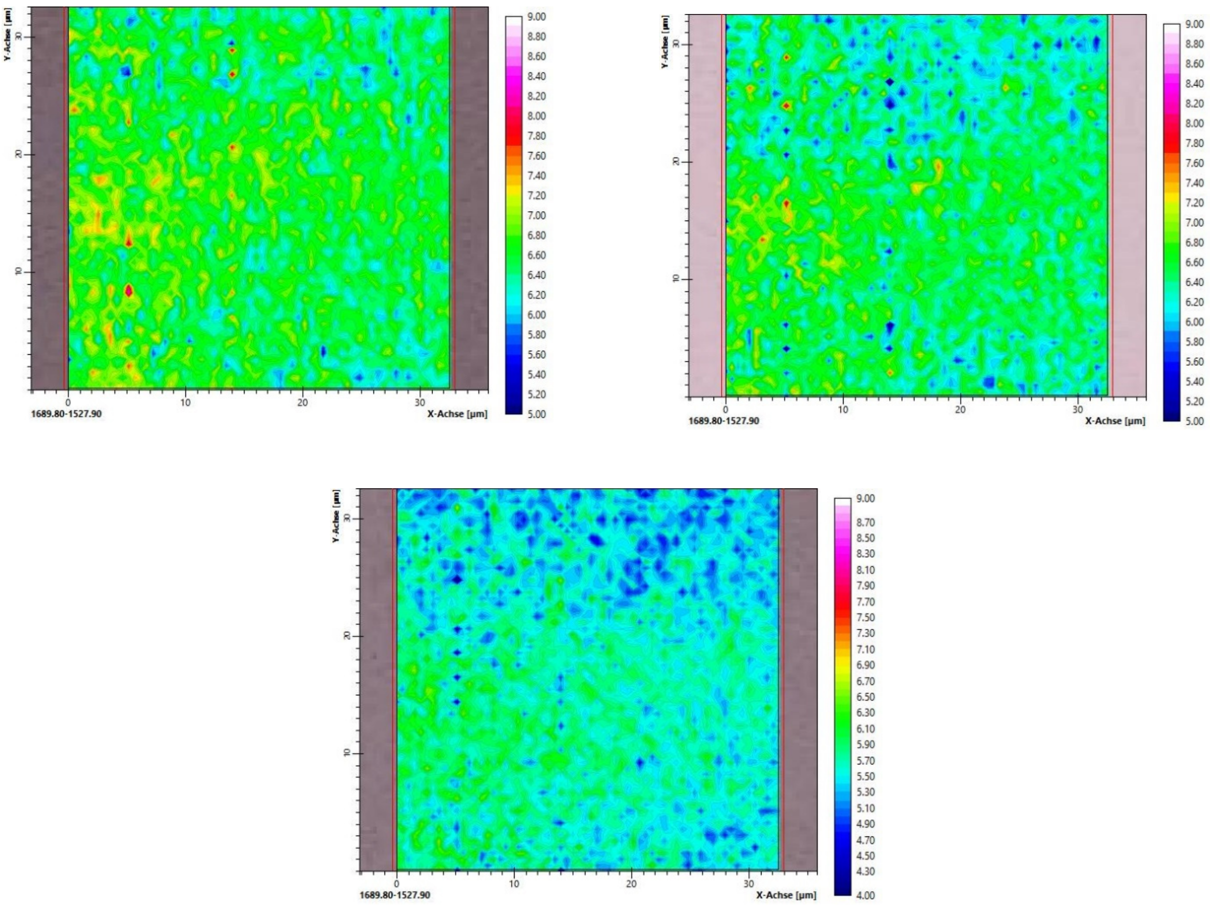

Figure S3. FTIR microspectral imaging of L_PEG after irradiation with an electron dose of 1x3 kGy (top left), 1x5 kGy (top right) and H_PEG after irradiation with an electron dose of 1x5 kGy (bottom).

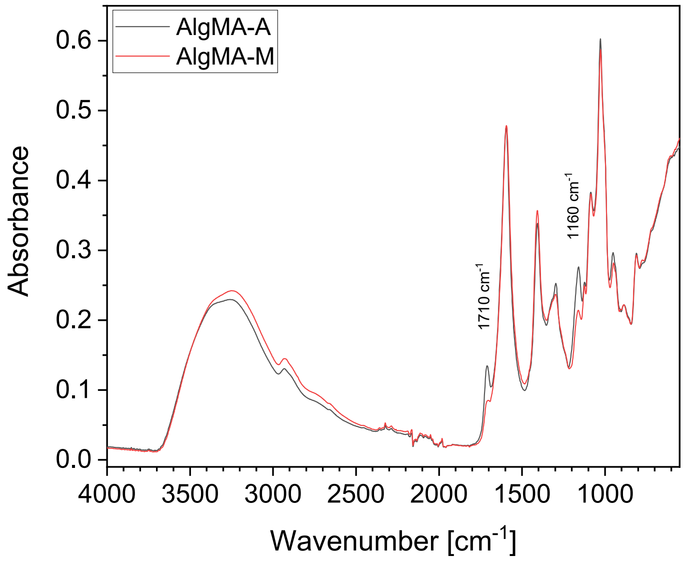

Figure S4. FTIR/ATR spectra of ALGMA H and ALGMA L after their functionalization.

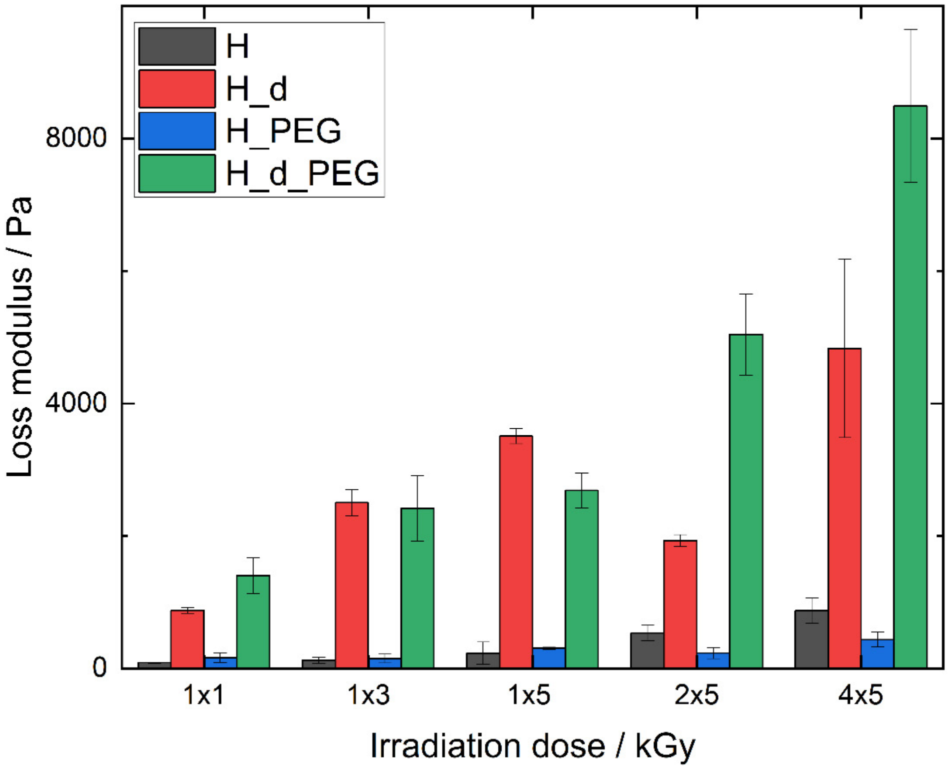

Figure S5. Loss modulus of AlgMA H hydrogels

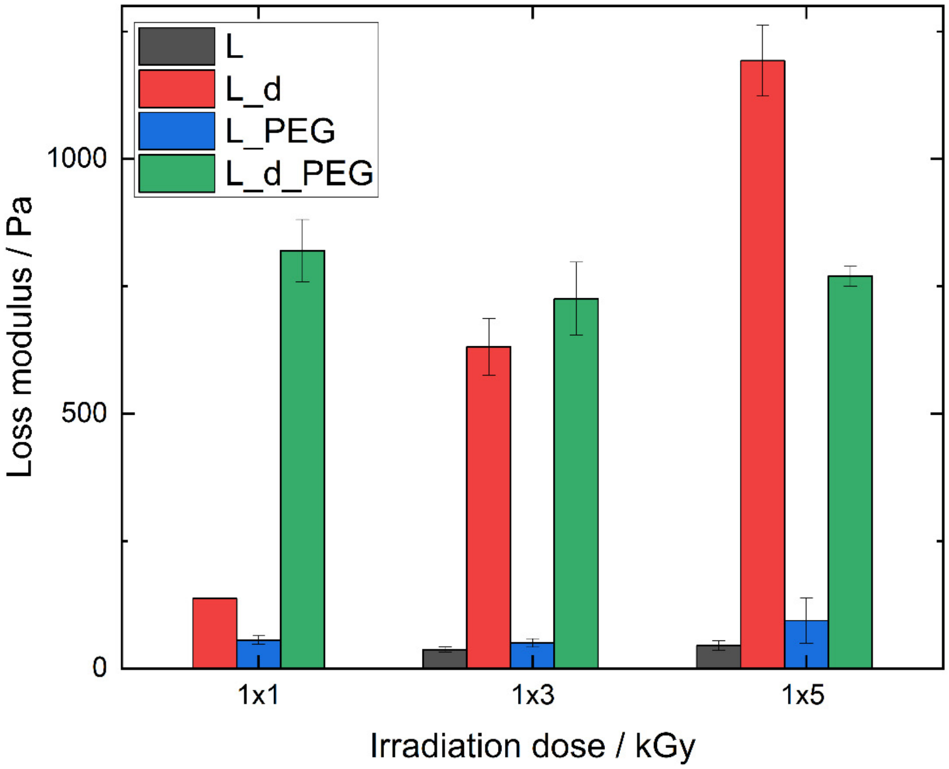

Figure S6. Loss modulus of AlgMA L hydrogels

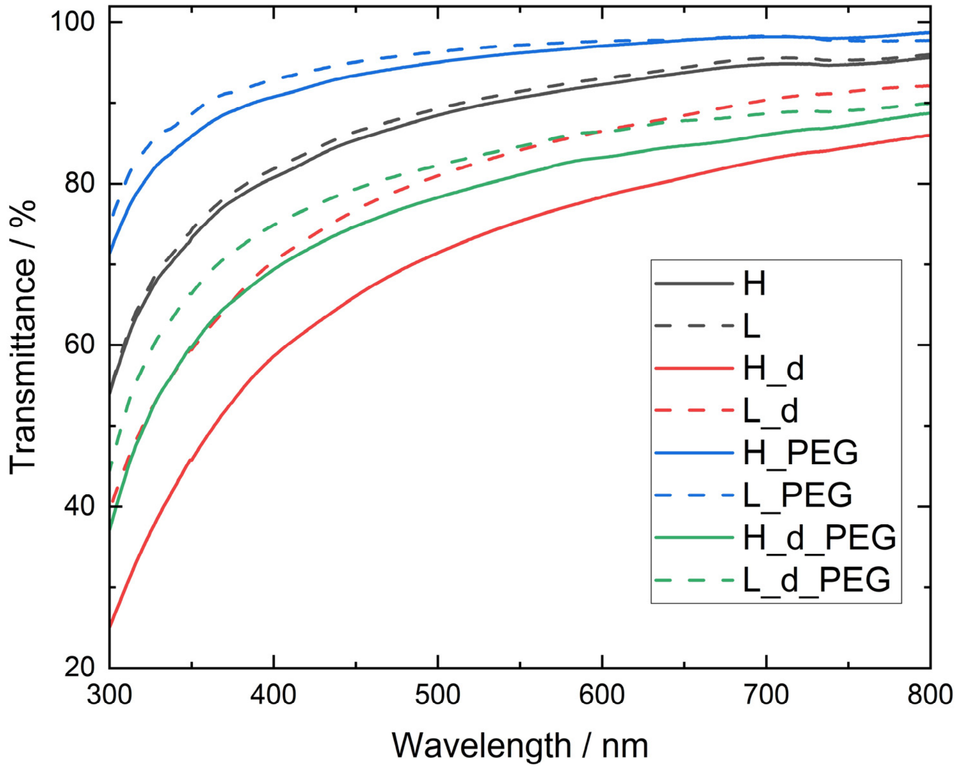

Figure S7. Frequency sweep with UV-VIS to measure the transparency of the polymers (1x5 kGy)

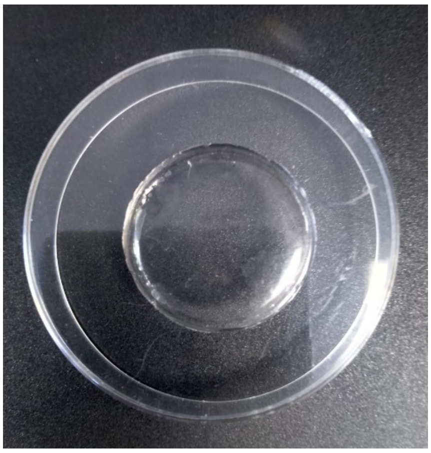

Figure S8. Visualization of the irradiated samples inside a petri dish indicating the transparency. The outer diameter of the petri dish is 55 mm.
